# Intermolecular-Interaction-Driven Adaptive Remodeling: A Network Perspective on Plant Abiotic Stress Responses

**DOI:** 10.3390/plants15121920

**Published:** 2026-06-22

**Authors:** Leidi Liu, Xiangfei Cheng, Yihua Xu, Lu Liu, Shuai Zhong, Xiaohua Chao, Yumin Chen, Chengde Yu, Chengming Fan, Changsong Zou

**Affiliations:** 1 Center for Molecular Interactions and Translational Applications, School of Life Sciences, Henan University, Kaifeng 475004, China; liuleidi@henu.edu.cn (L.L.); chengxf@henu.edu.cn (X.C.);; 2 State Key Laboratory of Cotton Bio-breeding and Integrated Utilization, School of Life Sciences, Henan University, Kaifeng 475004, China; 3 State Key Laboratory of Seed Innovation, Institute of Genetics and Developmental Biology, Chinese Academy of Sciences, Beijing 100101, China

**Keywords:** abiotic stress, adaptive remodeling, abscisic acid, reactive oxygen species, redox switch, calcium signaling, phosphorylation, chromatin regulation, stress memory, crop resilience

## Abstract

Abiotic stresses, including drought, salinity, alkalinity, temperature extremes, flooding, heavy metals, and emerging pollutants, challenge plant growth and productivity by disturbing water relations, ion balance, redox homeostasis, membrane stability, energy metabolism, and developmental progression. Although substantial progress has been made in the identification of stress-responsive hormones, second messengers, kinases, transcription factors, transporters, and metabolic regulators, plant stress adaptation cannot be fully explained by linear signaling cascades or single tolerance genes. A major unresolved question is how early molecular events are reorganized into coordinated physiological and developmental outputs that support survival, recovery, and productivity. In this review, we propose an intermolecular interaction-driven adaptive remodeling framework for plant abiotic stress responses. This framework emphasizes that stress tolerance emerges from dynamic changes in receptor–ligand recognition, protein–protein interactions, calcium decoding, redox-sensitive modification, phosphorylation networks, transcriptional regulation, chromatin-associated control, and metabolite-mediated feedback. We further emphasize ROS as integrative redox switches that connect stress sensing, defense activation, senescence-related transitions, and recovery, and chromatin-associated mechanisms as regulators that may stabilize primed or memory-like adaptive states. We discuss how these interaction networks converge on core signaling hubs, including abscisic acid, reactive oxygen species, Ca^2+^, and kinase/phosphatase systems, and how they remodel stomatal behavior, root architecture, ion and pH homeostasis, redox buffering, metabolism, development, and reproductive resilience. We further highlight how natural variation, multi-omics, genome editing, high-throughput phenotyping, and field validation can translate interaction-centered stress biology into crop resilience. This perspective provides a conceptual bridge between molecular stress perception, network behavior, physiological adaptation, and climate-resilient agriculture.

## 1. Introduction: Why Linear Stress–Response Models Are No Longer Sufficient

Plants grow in environments with continuous fluctuations in water availability, temperature, light intensity, soil salinity, nutrient status, oxygen supply, and pollutant exposure. Because plants are sessile organisms, they cannot avoid adverse conditions; instead, they must perceive environmental change and reorganize growth, metabolism, and development to maintain functionality. Abiotic stresses such as drought, salinity, alkalinity, heat, cold, flooding, heavy metals, and emerging pollutants affect plants through interconnected disturbances, including osmotic imbalance, ionic toxicity, redox disruption, membrane damage, protein instability, impaired photosynthesis, altered energy metabolism, and reproductive failure [1,2].

Over the past two decades, plant stress biology has identified many central components of abiotic stress responses. Abscisic acid (ABA) regulates drought, osmotic stress, seed dormancy, root growth, and stomatal movement [3,4]. Reactive oxygen species (ROS) are now recognized as essential signaling molecules and potential sources of oxidative injury [5,6,7]. Calcium ions act as rapid second messengers whose spatial and temporal signatures encode environmental information [8]. Protein kinases, phosphatases, transcription factors, ion transporters, antioxidant systems, metabolic enzymes, and chromatin regulators link stress perception to downstream physiological responses [2,9,10,11].

However, the traditional view of plant stress response as a linear cascade—from perception to signaling, gene expression, and adaptation—is increasingly insufficient. Many signaling components are shared across different stresses, yet plants generate stress-specific outputs. ABA, ROS, Ca^2+^, MAPKs, CDPKs, CBL–CIPK modules, transcription factors, and transporters are involved in multiple stress contexts, but their physiological consequences differ among drought, salinity, heat, cold, flooding, and metal toxicity [1,2,5,8,12]. These differences are shaped by signal timing, tissue type, developmental stage, subcellular localization, genetic background, and environmental combination [13,14].

Linear models also fail to capture the trade-offs inherent in stress adaptation. Protective responses consume energy, carbon skeletons, reducing power, nutrients, and developmental resources. Stomatal closure conserves water but limits CO_2_ uptake [15,16,17]. Ion extrusion and compartmentalization improve salt tolerance but require proton gradients and ATP [12,18,19,20,21,22,23]. Antioxidant and osmoprotective metabolism can prevent damage but may divert resources from growth and reproduction [5,7,24]. Thus, plants do not simply resist stress; they continuously rebalance survival, growth, reproduction, and recovery under resource limitation.

These limitations are particularly evident in crop improvement. Many genes that enhance stress resistance under controlled conditions do not consistently improve field productivity because field stress is dynamic, heterogeneous, and often combined [14,25,26,27,28]. Crop resilience should therefore be understood not as stress survival alone but as the ability to maintain productivity, quality, reproductive success, and recovery capacity under fluctuating environments [29,30,31,32,33].

**Figure 1:**
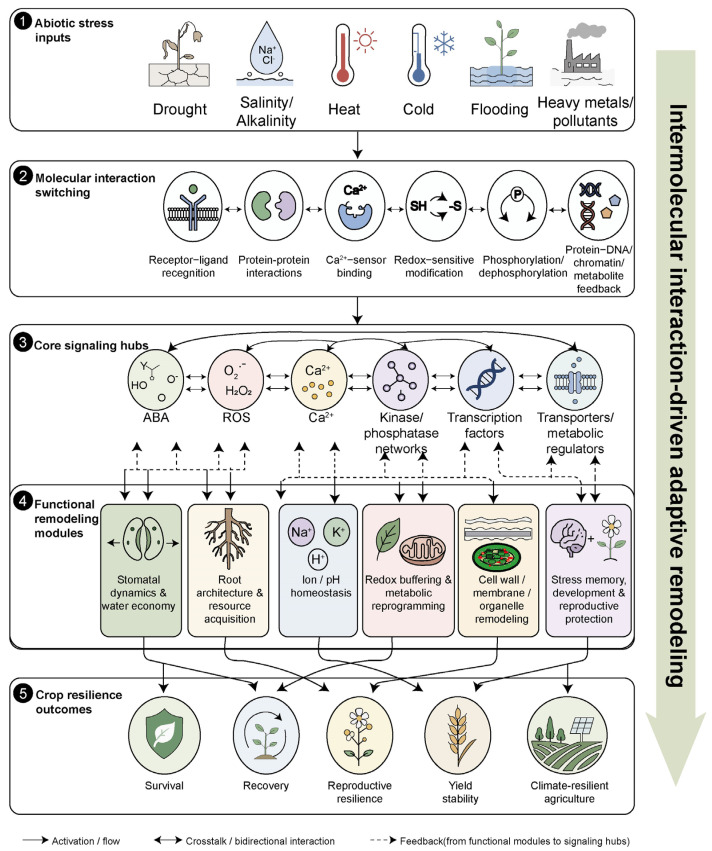
Intermolecular-interaction-driven adaptive remodeling model of plant abiotic stress responses.

In this review, we propose an intermolecular-interaction-driven adaptive remodeling framework for plant abiotic stress responses. We argue that stress adaptation emerges from dynamic changes in receptor–ligand recognition, protein–protein interactions, Ca^2+^ decoding, redox-sensitive modification, phosphorylation, transcriptional regulation, chromatin-associated control, transporter activity, and metabolite-mediated feedback. We first discuss molecular interactions as the primary language of stress signaling, then examine how ABA, ROS, Ca^2+^, and phosphorylation systems function as core signaling hubs. We next describe how these hubs remodel physiological functions and how shared modules are utilized under different stresses. Finally, we discuss how interaction-centered stress biology can guide crop improvement through natural variation, multi-omics, genome editing, phenotyping, and field validation. The overall logic of this framework is summarized in Figure 1.

## 2. Molecular Interactions as the Primary Language of Stress Signaling

Environmental stress information does not enter plant cells as an abstract signal. As outlined in Figure 1, it is first translated into changes in molecular states: receptors bind ligands, proteins associate or dissociate, ions interact with sensors, redox-active residues are modified, kinases phosphorylate substrates, transcription factors bind DNA, and metabolites reshape enzyme activity or signaling feedback. These interactions are the primary language through which plants perceive and process environmental change. A pathway-centered view can identify the components involved in stress responses, but an interaction-centered view explains how these components are assembled, modified, and redirected into adaptive network states [1,2].

This distinction is important because stress adaptation is not determined simply by the presence of a gene or protein. The same protein may have different effects depending on its interacting partners, localization, modification state, tissue context, and developmental timing. Likewise, the same stress signal may produce different outputs if it enters the network through different receptors, sensors, or feedback loops. Therefore, understanding plant abiotic stress responses requires attention not only to signaling components but also to the molecular interactions that connect them [2,13].

### 2.1. Receptor–Ligand Interactions Initiate Stress Information Flow

Receptor–ligand recognition provides one of the earliest layers of stress information processing. In hormone signaling, ligand binding can rapidly alter receptor conformation, recruit or inhibit downstream regulators, and redirect signaling outputs. The ABA receptor system is a representative example: ABA binding to PYR/PYL/RCAR receptors promotes receptor-mediated inhibition of clade A PP2C phosphatases, thereby allowing SnRK2 kinases to activate transcriptional and physiological responses [3,4,34,35]. This system illustrates how a small-molecule ligand can reorganize a protein interaction module.

Stress perception also involves extracellular and membrane-associated signaling. Peptide–receptor-like kinase systems can transmit information about cell-wall status, growth demand, tissue damage, or environmental change to intracellular signaling networks [36,37]. In salt stress, PAMP-INDUCED SECRETED PEPTIDE 3 (PIP3)-mediated signaling through RECEPTOR-LIKE KINASE 7 (RLK7) further illustrates how peptide–RLK modules can modulate stress tolerance [38]. Such systems are especially relevant at the cell wall–plasma membrane interface, where mechanical, osmotic, ionic, and apoplastic changes may be coupled to Ca^2+^ influx, ROS production, phosphorylation, and transcriptional responses.

Synthesis. Receptor–ligand interactions initiate stress information flow, but their output depends on receptor context, spatial organization, and downstream network state.

### 2.2. Protein–Protein Interactions Rewire Signaling Architecture

Protein–protein interactions are central to stress signal decoding. Many stress–response pathways are built from inducible or reversible interactions among receptors, kinases, phosphatases, transcription factors, transporters, scaffold proteins, E3 ubiquitin ligases, and metabolic enzymes. These interactions allow plants to rapidly modify signaling outputs without waiting for new transcription or translation [34,35,39,40].

ABA signaling again illustrates this principle. The adaptive output of ABA depends on dynamic interactions among ABA receptors, PP2Cs, SnRK2s, transcription factors, ion channels, and regulatory proteins. Salt stress involves interactions among calcium sensors, CBL-interacting protein kinases, Na^+^/H^+^ antiporters, K^+^ transporters, H^+^-ATPases, and 14-3-3 proteins [12,18,19,20]. Temperature responses involve interactions that regulate transcription-factor stability, heat-shock protein function, protein folding, ubiquitination, and phosphorylation [41,42,43].

Stress can rewire protein-interaction networks within seconds to minutes. Phosphorylation can create or disrupt docking sites, ubiquitination can direct regulators toward degradation, SUMOylation can alter transcription-factor activity, and changes in ion or redox state can modify protein conformation [10,11,44,45,46]. These rapid modifications allow plants to switch from growth-oriented networks to stress-protective networks.

Protein–protein interactions also help explain why single-gene manipulation often produces unstable outcomes. A gene may confer stress tolerance in one background but not another if its interacting partners differ. In polyploid crops, duplicated genes may have diverged in expression or interaction specificity. In different tissues, the same protein may join different complexes and produce distinct outputs [47,48,49,50]. Thus, the functional meaning of a protein depends on the network in which it operates.

Synthesis. Protein–protein interactions rewire signaling architecture and determine how stress information is distributed through regulatory networks. They provide mechanistic explanations for context-dependent responses and potential targets for precision crop improvement.

### 2.3. Protein–Ion Interactions Decode Calcium-Based Stress Information

Calcium ions are among the fastest and most versatile stress signals in plants. Drought, salinity, cold, heat, flooding, mechanical stimulation, and pathogen attack can all induce changes in cytosolic Ca^2+^ concentration. Yet these stresses do not produce identical outputs. Calcium specificity is generated not simply by the presence of Ca^2+^ but by the interaction between calcium signatures and calcium-decoding proteins [8,18,51].

Calcium signatures differ in amplitude, duration, frequency, subcellular localization, and propagation pattern. These signatures are interpreted by Ca^2+^-binding proteins such as calmodulins, calmodulin-like proteins, calcium-dependent protein kinases, and calcineurin B-like proteins. Once activated, these sensors interact with downstream kinases, phosphatases, ion channels, transcription factors, and transporters. In salt stress, calcium-dependent CBL–CIPK modules regulate ion transport and Na^+^ homeostasis. In guard cells, Ca^2+^ signals participate in ABA-regulated ion-channel activity and stomatal movement. In systemic signaling, calcium waves can transmit stress information from local perception sites to distant tissues [8,12,18,19].

The key issue is not whether Ca^2+^ is involved in stress signaling; it is how plants read Ca^2+^ information. Different cell types may express different calcium sensors, channels, pumps, and downstream targets. Therefore, identical or similar Ca^2+^ changes may be decoded differently in roots, guard cells, vascular tissues, mesophyll cells, or reproductive organs. This helps explain how Ca^2+^ can serve as a common stress signal while producing stress- and tissue-specific responses [18,19,51,52,53].

Synthesis. Calcium signaling is best understood as a protein–ion interaction system in which Ca^2+^ signatures are decoded by sensor networks to produce context-specific physiological outputs.

### 2.4. Redox-Sensitive Interactions Convert Oxidative Pressure into Information

ROS are produced in chloroplasts, mitochondria, peroxisomes, apoplasts, and plasma membrane-associated NADPH oxidase systems. Abiotic stresses often increase ROS production, but ROS should not be viewed only as damaging molecules. Controlled ROS production provides essential information for stress signaling, systemic communication, stomatal regulation, root development, acclimation, senescence-related transitions, and recovery [5,6,7]. Because many environmental cues converge on ROS, redox-sensitive interactions can act as early integrators rather than merely as markers of oxidative injury.

The biological meaning of ROS depends on redox-sensitive molecular interactions. ROS can oxidize cysteine residues, alter disulfide-bond formation, modify enzyme activity, regulate transcription factors, influence protein stability, and affect organellar communication. Such modifications can activate or inhibit signaling pathways and thereby couple oxidative pressure to phosphorylation, transcriptional control, metabolism, and stress-memory-related feedback. However, excessive ROS accumulation causes oxidative injury, damaging lipids, proteins, DNA, membranes, and photosynthetic structures [5,7,45].

Therefore, the central problem in ROS biology is not simply ROS production or ROS scavenging, but rather the maintenance of an information-rich redox state. Plants must preserve ROS signals long enough to trigger adaptation while preventing destructive accumulation. This balance is achieved through spatially controlled ROS generation and antioxidant systems, including superoxide dismutase, catalase, peroxidases, ascorbate, glutathione, thioredoxins, peroxiredoxins, flavonoids, and anthocyanins [24,46,54,55].

Synthesis. ROS acts as both a stress burden and signaling currency. Adaptive redox regulation depends on molecular interactions that preserve the informational value of ROS while preventing oxidative damage.

### 2.5. Protein–DNA, Chromatin-Associated, and Protein–Metabolite Interactions Stabilize Adaptive States

Early stress signaling is often rapid and reversible, but long-term adaptation requires more stable changes in gene expression, metabolism, development, and cellular state. Protein–DNA interactions, chromatin-associated regulation, and protein–metabolite interactions provide this longer-term stabilization [11,56,57,58,59,60,61,62,63,64].

Transcription factors bind stress-responsive promoters to activate or repress gene networks involved in osmotic adjustment, antioxidant defense, ion transport, hormone signaling, root development, stomatal regulation, and reproductive protection. However, transcription factor binding is not determined by the DNA sequence alone. It is influenced by chromatin accessibility, histone acetylation and methylation, DNA methylation, co-regulators, transcriptional feedback, and metabolite status [11,59,61,62].

Chromatin-based control is particularly relevant for stress priming and memory. Prior exposure to drought, heat, salinity, or oxidative stress may alter promoter accessibility or histone marks at selected stress-responsive loci, thereby lowering activation thresholds or accelerating transcriptional reactivation during subsequent stress. Such memory-like states can be beneficial when stress recurs, but they may also impose growth or reproductive costs if stress programs remain active after stress release [60,61,62,63,64].

Metabolites also function as regulatory molecules rather than merely downstream products. ABA, malate, sugars, proline, amino acids, organic acids, glutathione, flavonoids, anthocyanins, lipids, and secondary metabolites can affect osmotic balance, redox buffering, enzyme activity, stomatal movement, pH regulation, and signaling feedback [15,24,44,55].

Synthesis. Protein–DNA, chromatin-associated, and protein–metabolite interactions convert early stress signals into durable but reversible adaptive states, and their value depends on developmental timing, memory stability, and growth cost.

## 3. Core Signaling Hubs and Their Crosstalk

Although abiotic stresses trigger many molecular interactions, a limited number of signaling hubs appear repeatedly across stress contexts. As shown in Figure 1, ABA, ROS, Ca^2+^, and phosphorylation-based kinase/phosphatase networks are especially important because they integrate multiple forms of environmental information and connect early perception with downstream physiological outputs [2,3,5,8,9]. These hubs are not isolated pathways. They form a dense regulatory network in which hormone signaling, redox state, calcium dynamics, phosphorylation, ion transport, transcription, metabolism, and development continuously influence one another [2,13].

A hub-based view helps explain two major features of plant stress biology. First, different stresses often activate the same components. Drought, salinity, heat, cold, flooding, and heavy metals can all involve ROS, Ca^2+^, kinase activation, and hormonal changes. Second, shared components can generate different outputs. This specificity emerges from differences in signal timing, amplitude, localization, cell type, interacting partners, feedback loops, and developmental context [1,2,52,53].

### 3.1. ABA-Centered Regulatory Circuits: Beyond a Drought Hormone

ABA is one of the most intensively studied regulators of plant abiotic stress responses. It plays major roles in drought, osmotic stress, seed dormancy, root growth, stomatal movement, ion transport, and stress-responsive gene expression. The canonical ABA pathway is built around PYR/PYL/RCAR receptors, clade A PP2C phosphatases, and SnRK2 kinases [3,4]. ABA binding promotes receptor-mediated inhibition of PP2Cs, allowing SnRK2 activation and subsequent regulation of transcription factors, ion channels, and other downstream proteins [34,35].

This core pathway is essential, but it is not sufficient to explain ABA output in real biological contexts. ABA dynamics are shaped by biosynthesis, catabolism, transport, conjugation, local concentration, receptor composition, and tissue-specific sensitivity. Long-distance ABA movement can connect root perception with shoot responses, while local ABA accumulation can regulate guard-cell behavior, root growth, and stress-responsive transcription [4,65,66].

ABA also interacts extensively with other signaling systems. In guard cells, ABA-induced stomatal closure requires ROS production, Ca^2+^ signaling, ion-channel regulation, pH changes, cytoskeletal remodeling, and metabolic adjustment [6,15,16,36]. In roots, ABA interacts with auxin, ethylene, brassinosteroids, peptide signaling, ROS, Ca^2+^, and nutrient status to regulate growth under stress [37,67]. Light signaling and circadian regulation can alter ABA sensitivity, while energy and metabolic status influence ABA outputs [68,69].

These context-dependent outputs make ABA a powerful but potentially costly regulatory hub. Strong ABA activation can conserve water, but prolonged or constitutive activation may restrict photosynthesis, growth, seedling establishment, or yield [39,50].

Synthesis. ABA is not merely a drought hormone. It is a modular signaling hub whose output depends on receptor composition, phosphatase–kinase balance, transport, localization, crosstalk, and growth–defense trade-offs.

### 3.2. ROS as Signal, Stress Burden, Network Amplifier, and Redox Switch

ROS are central to plant stress responses because they connect environmental disturbance with signaling, metabolism, organellar function, defense activation, senescence, and damage control. Drought, salinity, heat, cold, heavy metals, flooding, high light, pollutants, and pathogen attack can all stimulate ROS production. This broad convergence makes ROS a reusable redox language through which plants integrate different environmental inputs. However, ROS biology is defined by a paradox: ROS are required for adaptation, but excessive ROS accumulation causes injury [5,6,7].

Controlled ROS production can activate stress-responsive genes, regulate stomatal movement, modulate root development, promote systemic signaling, and trigger acclimation. ROS generated by NADPH oxidases, chloroplasts, mitochondria, peroxisomes, and apoplastic enzymes have different spatial and temporal properties. Source, amplitude, duration, diffusion range, and subcellular localization determine whether ROS function as local signals, systemic signals, network amplifiers, senescence-associated switches, or damage factors [5,7,49].

ROS also interact with ABA and Ca^2+^. ABA can induce ROS production in guard cells; ROS can promote Ca^2+^ influx or release, and Ca^2+^ can activate ROS-generating enzymes. These feedback loops amplify or stabilize stress signals and help connect early perception with stomatal closure, root-system remodeling, transcriptional activation, and systemic communication [6,36,46]. In addition, ROS can regulate protein activity through redox-sensitive modifications, thereby linking oxidative state with phosphorylation, transcription, metabolism, organellar communication, and chromatin-associated responses [45].

Antioxidant systems are therefore not simply protective barriers. They shape the amplitude, duration, and localization of ROS signals. A highly active antioxidant system may prevent damage but also suppress useful signaling if ROS are removed too quickly. Conversely, weak antioxidant capacity may allow ROS signals to become destructive. Plant resilience therefore requires neither maximal ROS scavenging nor uncontrolled ROS production, but maintenance of an information-rich redox state that activates adaptive networks while preventing oxidative injury [24,46,54,55].

Synthesis. ROS functions as signaling molecules, stress burdens, redox switches, and network amplifiers. Their adaptive value depends on whether plants can maintain ROS within an information-rich but non-destructive range and coordinate ROS dynamics with ABA, Ca^2+^, phosphorylation, metabolism, senescence, and recovery.

### 3.3. Calcium Signatures and Decoding Networks

Ca^2+^ signaling provides a rapid mechanism for converting environmental change into cellular information. Many abiotic stresses induce Ca^2+^ transients, but specificity is generated by the shape and location of Ca^2+^ signatures and by the sensor networks that decode them [8,18,51].

Calcium-decoding proteins include calmodulins, calmodulin-like proteins, calcium-dependent protein kinases, and CBL proteins. These sensors interact with kinases, channels, pumps, transcriptional regulators, and transporters. Through these interactions, Ca^2+^ signals regulate stomatal movement, salt-induced ion transport, root growth, cold responses, heat responses, and systemic signaling [8,19,51].

Salt stress provides a clear example of calcium-based decoding. Salt-induced Ca^2+^ changes can activate CBL–CIPK modules that regulate Na^+^ extrusion, K^+^ retention, and ion homeostasis. In guard cells, Ca^2+^ participates in ABA-regulated ion-channel control and stomatal closure. In long-distance signaling, Ca^2+^ waves can transmit local stress perception to distant organs [8,12,18,19].

A major unresolved question is how Ca^2+^ specificity is generated in different cell types. Whole-organ measurements cannot distinguish the signatures produced in root epidermis, cortex, endodermis, xylem-associated cells, guard cells, mesophyll cells, or reproductive tissues. Cell-type-specific biosensors and spatially resolved analyses will be essential for determining how plants convert shared Ca^2+^ signals into distinct adaptive outputs [52,53].

Synthesis. Ca^2+^ is a reusable second messenger whose specificity emerges from signature dynamics and sensor-network context rather than from calcium elevation alone.

### 3.4. Kinase/Phosphatase Networks and Post-Translational Control

Protein phosphorylation is a major mechanism through which stress information is transmitted and amplified. SnRK2s, MAPKs, CDPKs, CBL–CIPKs, RAF-like kinases, receptor-like kinases, and phosphatases form interconnected networks that regulate hormone signaling, ion transport, transcription, metabolism, and development [9,10,40].

SnRK2 kinases are central in ABA and osmotic stress signaling. They connect receptor-mediated ABA perception with downstream transcription factors and ion channels. RAF-like kinases can activate SnRK2 modules under osmotic stress, suggesting that ABA-dependent and ABA-independent signaling are integrated through phosphorylation cascades [35,40,70]. PP2C phosphatases attenuate ABA signaling and provide negative regulation, but their activity is itself controlled by receptor interaction and feedback [34,39].

CBL–CIPK modules are particularly important in salt and ion homeostasis. Calcium sensors recruit and activate CIPKs, which regulate transporters and pumps involved in Na^+^ extrusion, K^+^ uptake, and pH regulation. CDPKs connect Ca^2+^ signals with phosphorylation of downstream proteins. MAPK cascades contribute to stress-responsive transcription, ROS signaling, hormone crosstalk, and developmental adjustment [18,19,21,51].

Post-translational control extends beyond phosphorylation. Ubiquitination regulates protein stability; SUMOylation can modify transcription-factor activity and stress tolerance; acetylation and other chromatin-associated modifications influence transcriptional states; redox modifications alter protein activity; and proteolysis resets signaling networks [11,42,44,45]. Together, these processes allow plants to modify existing proteins rapidly and reversibly, which is essential during the early stress responses.

Because these modifications are rapid and reversible, post-translational regulation provides a major source of signaling flexibility. It determines whether early stress perception is converted into transient signaling, sustained acclimation, or growth-costly defense activation [10,11,44,45].

Synthesis. Kinase/phosphatase and post-translational networks function as signal-decoding and signal-tuning systems.

### 3.5. Hormonal Crosstalk and Energy-State Integration

Although ABA is central, plant stress adaptation involves multiple hormones. Auxin, ethylene, jasmonates, salicylic acid, brassinosteroids, gibberellins, cytokinins, strigolactones, and peptide hormones influence root architecture, stomatal development, growth inhibition, senescence, defense, flowering, and reproductive resilience [13,37,43,67].

Hormonal crosstalk is essential because stress adaptation requires prioritization. Under drought, plants may reduce leaf expansion but maintain their root growth. Under salt stress, ethylene and auxin may reshape root architecture. Under heat, brassinosteroids and heat-shock pathways may interact to regulate thermotolerance. Under flooding, ethylene contributes to hypoxia responses and morphological adjustment. In each case, hormonal networks help determine whether the plant prioritizes growth, maintenance, protection, escape, dormancy, reproduction, or recovery [13,37,43,71].

Energy-state integration adds another layer. Stress responses require ATP, reducing power, carbon skeletons, nitrogen assimilation, and metabolic flexibility. Energy sensors and metabolic feedback can influence hormone sensitivity, transcription, autophagy, respiration, and growth. A stress response that is protective but energetically unsustainable may reduce yield. Therefore, signaling hubs must be understood together with resource allocation [39,44,45].

Synthesis. Hormonal crosstalk and energy-state integration determine how plants choose among competing adaptive priorities. They connect stress signaling with growth, development, reproduction, and yield formation.

## 4. Functional Remodeling Modules

Stress signaling becomes biologically meaningful only when it is translated into functional outputs. ABA accumulation, ROS bursts, Ca^2+^ transients, phosphorylation cascades, and transcriptional activation do not by themselves define stress tolerance. In the model summarized in Figure 1, their adaptive value depends on whether they reorganize plant physiology in ways that preserve water status, maintain ion balance, protect cellular structures, sustain metabolism, and secure reproductive success [1,2].

These outputs are not independent endpoints. Stomatal movement influences photosynthesis and leaf temperature; root architecture determines water and nutrient acquisition; ion transport reshapes osmotic balance and cellular pH; redox buffering controls both damage prevention and signal propagation; metabolic reprogramming supports osmotic adjustment and energy redistribution; and developmental regulation determines whether plants prioritize survival, growth, reproduction, or recovery. In this sense, stress adaptation is an integrated reconfiguration of multiple functional systems.

### 4.1. Stomatal Dynamics and Water Economy

Stomata form a central functional interface between stress signaling and whole-plant water economy. By controlling CO_2_ uptake and transpirational water loss, they influence photosynthesis, leaf temperature, hydraulic status, and water-use efficiency. Stress-regulated stomatal behavior depends not only on steady-state conductance but also on the timing, sensitivity, reversibility, and environmental responsiveness of guard-cell movement [6,15,16,17].

Guard-cell signaling integrates ABA, ROS, Ca^2+^, nitric oxide, ion channels, malate metabolism, mitochondrial function, light signaling, and redox regulation [6,15,16,17,36,69,72,73]. This integration allows stomata to respond to drought, fluctuating light, vapor pressure deficit, CO_2_, pathogens, and metabolic state. Therefore, stomata should not be viewed as simple ABA-controlled valves but as dynamic regulatory units that process multiple internal and external signals.

For crop resilience, the key trait is not necessarily lower conductance but improved stomatal kinetics. Faster closure under sudden stress, efficient reopening after stress release, and coordination with photosynthetic capacity can influence biomass, canopy temperature, water-use efficiency, and yield stability [17,29,30,31,32,33,74].

Synthesis. Stomatal adaptation is a dynamic water-economy regulation rather than static closure.

### 4.2. Root Architecture and Resource Acquisition

Roots are primary organs for sensing soil water status, salinity, alkalinity, nutrient availability, mechanical resistance, and rhizosphere signals. Stress-induced changes in primary root growth, lateral root formation, root hair development, cortical anatomy, hydraulic conductivity, and root-to-shoot communication strongly influence water and nutrient acquisition [67,75,76,77].

Root remodeling is regulated by hormonal and signaling crosstalk involving ABA, auxin, ethylene, brassinosteroids, peptide signaling, ROS, and Ca^2+^ [13,37,67]. However, the adaptive value of a root phenotype is context-dependent. Deeper roots may improve water acquisition under terminal drought but may not benefit crops under shallow intermittent rainfall. Lateral root proliferation may enhance nutrient foraging but can increase exposure to saline or toxic soil zones. Thus, “more roots” are not always better.

For crop translation, root traits should be evaluated functionally rather than descriptively. The key question is whether root architecture improves resource acquisition, ion balance, recovery, and yield under realistic soil and stress conditions [21,23,75,76,78].

Synthesis. Root-system remodeling is a context-dependent resource-acquisition strategy whose value depends on soil environment, carbon cost, and shoot demand.

### 4.3. Ion and pH Homeostasis

Ion and pH homeostasis is essential under salinity, alkalinity, nutrient imbalance, and metal toxicity. Salinity imposes osmotic stress and ion toxicity, whereas alkalinity additionally disrupts apoplastic pH, nutrient availability, proton gradients, and membrane transport. Maintaining cytosolic K^+^/Na^+^ balance, vacuolar sequestration, membrane potential, and pH stability is therefore critical for enzyme activity, photosynthesis, and growth [12,18,19,20,21,22,23,78,79,80,81,82,83].

This module depends on coordinated transport and energy supply. Na^+^/H^+^ antiporters, HKT-type transporters, HAK/KUP/KT transporters, H^+^-ATPases, vacuolar transport systems, and aquaporins contribute to ion partitioning and water balance [12,18,19,20,21,78,79,80,81]. Crop studies provide additional support for transporter-centered ion-homeostasis modules. In cotton, GhNHX1 positively contributes to salt tolerance, whereas C2-domain ABA-related proteins regulate the dynamics of the plasma membrane H^+^-ATPase AHA1 under alkali stress, linking transporter activity, membrane-localized regulation, and pH homeostasis [84,85]. However, ion extrusion, sequestration, and pH regulation require ATP and proton gradients. Thus, ion homeostasis is not only a transport problem but also an energy-allocation problem.

Cell-type-specific transport and natural variation provide strong evidence that salt tolerance depends on regulatory context rather than transporter abundance alone [21,22,23,78,84,85]. Crop improvement should therefore prioritize tissue-specific ion partitioning and energy-efficient regulation.

Synthesis. Ion and pH homeostasis represent an energy-dependent module that coordinates transport, signaling, and metabolic capacity.

### 4.4. Redox Buffering and Metabolic Reprogramming

Redox and metabolic remodeling connect stress signaling with cellular protection and resource allocation. Under drought, salinity, heat, cold, heavy metals, and flooding, plants must maintain ROS within an information-rich but non-destructive range while adjusting carbon, nitrogen, lipid, and secondary metabolism [5,7,24,46,54]. In contrast to Section 3.2, which focuses on ROS as a signaling hub, this section emphasizes buffering capacity, metabolic allocation, and recovery cost.

Metabolites such as malate, soluble sugars, organic acids, proline, glutathione, flavonoids, anthocyanins, and lipids function as osmoprotectants, antioxidants, pH regulators, structural components, and feedback signals [15,24,44,55]. Redox-buffering modules also interact with transcriptional and structural regulation. AtMYB49 modulates salt tolerance through cuticle formation and antioxidant defense, whereas cotton GhWRKY207 promotes drought tolerance by activating antioxidant-related genes such as GhCSD3 and GhFSD2 [57,58]. However, metabolic protection is costly. Osmolyte production, antioxidant regeneration, detoxification, and repair consume carbon skeletons, ATP, reducing power, and nutrients.

Therefore, resilience depends not on maximal accumulation of protective metabolites but on efficient and reversible metabolic allocation. The most adaptive genotypes may be those that preserve signaling, limit injury, and recover rapidly after stress release [29,30,33].

Synthesis. Redox and metabolic remodeling allow plants to preserve signaling, prevent damage, and redistribute resources without excessive growth cost.

### 4.5. Cell Wall, Membrane, and Organelle Remodeling

Stress adaptation is constrained by cellular architecture. The cell wall, plasma membrane, endomembrane system, chloroplasts, mitochondria, and peroxisomes act as both stress targets and signaling platforms [15,37,45]. Cell-wall remodeling can modify growth, water retention, ion binding, and mechanical resistance, while membrane remodeling influences fluidity, permeability, transporter activity, and signal compartmentalization.

Salt-induced organellar responses also illustrate the structural dimension of stress signaling. In Arabidopsis root cells, NADPH oxidase-derived ROS promote mitochondrial alkalization under salt stress, providing a direct link between plasma membrane ROS production, organellar pH dynamics, and cellular stress adaptation [59]. Organelles also contribute through energy metabolism, ROS production, protein quality control, and retrograde signaling. Chloroplasts are especially sensitive to drought, heat, salt, and high light because stress affects photosynthetic electron transport. Mitochondria adjust respiration and energy production, whereas peroxisomes contribute to ROS metabolism, photorespiration, and lipid-derived signaling [5,15,45].

Structural remodeling can enhance protection but may also restrict growth. Therefore, physical cellular architecture should be considered an active component of adaptive remodeling rather than a passive target of stress injury.

Synthesis. Cell walls, membranes, and organelles provide the physical platforms on which stress-signaling networks are assembled and executed.

### 4.6. Stress Memory, Epigenetic Remodeling, Developmental Adjustment, and Reproductive Protection

Stress adaptation operates across multiple time scales. Rapid responses involve ion fluxes, ROS production, Ca^2+^ transients, and phosphorylation, whereas longer-term acclimation may involve transcriptional reprogramming, chromatin remodeling, metabolic adjustment, and developmental changes [11,60,61,62,63,64]. Stress memory may allow plants exposed to prior stress to respond more rapidly or effectively to later stress, but its stability and agronomic value remain context-dependent.

Epigenetic and chromatin-based mechanisms can contribute to stress memory by modifying chromatin accessibility, histone acetylation or methylation, DNA methylation, and the responsiveness of stress-associated promoters. Through priming, a prior stress exposure may lower the activation threshold of defense-related genes or accelerate their reactivation during subsequent drought, heat, salinity, or oxidative stress. However, stress memory is not automatically beneficial. Persistent activation of stress programs may impose growth or reproductive costs, and not all memory states are stable, reversible, or heritable [60,61,62,63,64].

Developmental adjustment is another major output of stress networks. Plants may alter leaf expansion, senescence, root-to-shoot ratio, flowering time, reproductive development, and seed filling. These responses can support survival but may also reduce yield potential if they disrupt source–sink balance or reproductive timing.

Reproductive resilience deserves particular attention because flowering, pollen development, fertilization, seed set, grain filling, fruit formation, and fiber quality are often more stress-sensitive than vegetative survival [11,29,43,86,87,88,89,90,91]. Therefore, stress tolerance at the seedling stage should not be assumed to predict crop resilience. The agronomic value of stress memory and priming should be judged by recovery, reproductive success, and yield stability rather than by survival alone.

Synthesis. Stress memory, epigenetic remodeling, and developmental adjustment extend adaptation beyond immediate survival, but their value depends on reversibility, recovery, reproductive success, and yield stability.

## 5. Stress-Specific Deployment of Shared Modules

Although drought, salinity, heat, cold, heavy metals, flooding, and other stresses impose distinct challenges, they recruit many shared signaling components. ABA, ROS, Ca^2+^, protein kinases, phosphatases, transcription factors, transporters, and metabolic regulators are repeatedly activated across stress types. The central question is therefore not whether these components are involved, but how they are deployed differently to generate stress-specific outputs [1,2,5,8]. This stress-specific deployment of shared modules forms the stress-context layer of the adaptive remodeling model (Figure 1).

Stress specificity arises from several sources: the nature of the primary disturbance, the tissue in which the signal is perceived, the duration and intensity of stress, the developmental stage of the plant, and the interaction among signaling modules. Shared signaling hubs are best understood as reusable regulatory modules whose outputs depend on network context.

### 5.1. Drought Stress: Hydraulic Limitation and Stomatal–Root Coordination

Drought primarily challenges plant water relations by reducing soil water availability, lowering plant water potential, limiting cell expansion, restricting photosynthesis, and increasing hydraulic risk [1,2]. Drought adaptation therefore requires coordination among ABA signaling, stomatal behavior, root architecture, hydraulic regulation, antioxidant defense, osmotic adjustment, and recovery capacity [3,4,6,15,16,17,65].

The major trade-off is water conservation versus carbon gain. ABA-mediated stomatal closure reduces transpirational water loss but limits CO_2_ assimilation. Root growth can improve water acquisition but requires carbon investment. Osmotic and antioxidant responses protect cells but consume metabolic resources. Therefore, drought resilience depends on coordinated whole-plant adjustment rather than isolated activation of drought-responsive genes.

In agriculture, drought is often intermittent and combined with heat or nutrient limitation. Resilience should therefore be evaluated through water-use efficiency, stomatal kinetics, root water capture, reproductive stability, recovery after rewatering, and yield maintenance rather than survival alone [29,30,31,32,33,74].

### 5.2. Salinity and Alkalinity: Ion Toxicity, Osmotic Stress, and pH Disturbance

Salinity and alkalinity impose overlapping but distinct constraints. Salinity causes osmotic stress and ion toxicity, especially through excess Na^+^ and Cl^−^, whereas alkalinity additionally disrupts pH homeostasis, nutrient availability, proton gradients, and root-zone chemistry [12,22]. Plants under saline–alkaline conditions must manage water deficit, ionic imbalance, oxidative stress, and pH disturbance simultaneously.

Salt tolerance depends on coordinated Na^+^ exclusion, K^+^ retention, vacuolar sequestration, H^+^-ATPase activity, aquaporin regulation, root architecture, and energy allocation [12,18,19,20,21,22,23,78,79,80,81,84,85]. In wheat, the domestication-related Q factor negatively regulates salt tolerance by repressing TaSOS1 and ROS-scavenging genes, providing a crop example in which ion-homeostasis and redox modules are transcriptionally coupled [92]. Alkalinity places additional demand on proton pumps and pH-regulatory systems because external high pH can reduce nutrient solubility and disrupt proton-coupled transport [19,55].

The key challenge is maintaining homeostasis without unsustainable metabolic cost. Tissue-specific transporter regulation and natural variation illustrate how ion-homeostasis modules can be tuned for crop improvement [21,22,23,78,84,85].

### 5.3. Heat Stress: Protein Stability, Photosynthetic Protection, and Reproductive Vulnerability

Heat stress destabilizes proteins, increases membrane fluidity, impairs photosynthesis, accelerates respiration, promotes ROS accumulation, and disrupts reproductive development. Protein quality control through HSF–HSP networks is central, but heat adaptation also requires redox regulation, chloroplast protection, membrane remodeling, hormonal crosstalk, and metabolic adjustment [11,43,45,93].

A major agronomic concern is that reproductive tissues are often more heat-sensitive than vegetative tissues. Heat-induced disruption of pollen development, fertilization, seed set, grain filling, and reproductive-stage physiology can become a major yield bottleneck [29,86,87,88,89,90,91]. Thus, seedling thermotolerance should not be assumed to predict reproductive-stage heat resilience.

Heat stress frequently coincides with drought. Drought-induced stomatal closure conserves water but can reduce transpirational cooling, illustrating how single-stress responses may become maladaptive under combined stress [14,25,26,27,28].

### 5.4. Cold Stress: Membrane Stability, Transcriptional Reprogramming, and Acclimation

Cold stress reduces membrane fluidity, slows enzymatic reactions, disrupts photosynthesis, and increases oxidative stress. Cold acclimation involves transcriptional, metabolic, and structural remodeling, with the ICE–CBF–COR pathway serving as a central regulatory module [41,42,59].

Cold tolerance also depends on protein stability, ubiquitination, lipid remodeling, osmoprotection, antioxidant defense, and developmental timing [41,42,59,94]. Natural promoter variation affecting cold tolerance during tomato domestication illustrates how regulatory variation can contribute to stress adaptation without necessarily relying on constitutive stress activation [94].

The major trade-off is cold protection versus growth and phenological adaptation. Enhancing cold resilience must therefore be coordinated with developmental timing and yield potential.

### 5.5. Heavy Metals and Emerging Pollutants: Detoxification Beyond Antioxidant Defense

Heavy metals and emerging pollutants disrupt protein function, nutrient balance, root development, photosynthesis, and redox homeostasis. Toxic elements such as cadmium, arsenic, chromium, copper, and lead can also accumulate in edible tissues, linking plant stress biology with food safety [54,95].

Metal tolerance involves uptake restriction, chelation, sequestration, efflux, antioxidant defense, and repair. However, tolerance should not be reduced to antioxidant capacity. Metals also affect nutrient competition, transporter specificity, rhizosphere chemistry, and long-distance partitioning [95].

Emerging pollutants such as microplastics, nanoparticles, and pharmaceutical residues add further complexity by interacting with soil structure, microbial communities, water retention, and metal mobility. Future work should therefore integrate plant physiology with soil chemistry, rhizosphere ecology, and food-safety assessment [14,25,95].

### 5.6. Flooding and Waterlogging: Oxygen Deficiency and Energy Reconfiguration

Flooding and waterlogging primarily limit oxygen availability in roots. Hypoxia or anoxia restricts aerobic respiration, reduces ATP production, alters redox state, and affects nutrient uptake [71]. Unlike drought, flooding creates excess water but restricts oxygen diffusion, showing that opposite environmental conditions can both disrupt energy balance.

Adaptive responses include aerenchyma formation, adventitious root development, anaerobic metabolism, carbohydrate management, ethylene signaling, and recovery from reoxygenation stress [71]. Because reoxygenation can trigger oxidative damage, flooding tolerance requires both hypoxia survival and post-flood recovery.

For crop improvement, relevant traits include root aeration, carbohydrate reserve maintenance, adventitious root formation, recovery after drainage, and compatibility with field management.

### 5.7. Combined Stresses: Emergent Network States Under Field Conditions

In natural and agricultural environments, plants rarely encounter a single stress in isolation. Drought often coincides with heat; salinity may combine with alkalinity or nutrient imbalance; flooding may be followed by reoxygenation stress; and pollutants may interact with metals or soil chemistry. Such combinations can produce responses distinct from those induced by each stress individually [14,25,26,27,28].

Combined stresses challenge shared signaling hubs in conflicting ways. Drought promotes stomatal closure, whereas heat may require stomatal opening for transpirational cooling. Salt stress demands ion exclusion and osmotic adjustment, whereas nutrient deficiency may require altered transporter activity. Flooding tolerance requires hypoxia survival, but drainage and reoxygenation can trigger oxidative bursts. These conflicts support the adaptive remodeling framework, in which plants generate emergent network states rather than simply adding single-stress responses.

Gene-level studies further support this view. HSF-type regulators such as HsfB2b/SlHSFB2b and NAC-type regulators such as IbNAC3 can be interpreted as combined-stress regulatory nodes because they connect heat-shock protein accumulation, ROS homeostasis, osmotic adjustment, membrane protection, and developmental regulation. HsfB2b-type regulation has been associated with acquired thermotolerance through modulation of heat-inducible HSF expression [96], whereas NAC-type regulators often integrate ABA- and ROS-responsive transcription with drought, salinity, and recovery-related responses. Such examples illustrate why combined-stress tolerance is better understood as coordinated module balancing than as maximal activation of isolated stress pathways.

Field resilience therefore depends on the ability to reorganize shared signaling modules under complex, fluctuating environments. Future experiments should include gradual stress imposition, stress combinations, recovery periods, and reproductive-stage exposure. Gene function should be evaluated not only through survival assays but also through stomatal kinetics, canopy temperature, ion partitioning, antioxidant capacity, fertility, post-stress recovery, and yield stability.

## 6. From Mechanism to Crop Resilience

Mechanistic studies have identified many stress-responsive receptors, kinases, transcription factors, transporters, antioxidant enzymes, metabolic regulators, and chromatin-associated factors. However, many genes that improve stress resistance under controlled conditions do not produce stable yield benefits in the field. This gap reflects a central problem: crop resilience is not equivalent to stress survival [29,30,31,32,33]. This translational gap represents the crop-resilience layer of the framework shown in Figure 1.

### 6.1. The Translational Gap: Survival Is Not Resilience

Laboratory stress assays often measure seedling survival, electrolyte leakage, chlorophyll content, root length, ion accumulation, or short-term biomass retention. These traits are useful for mechanistic analysis but do not necessarily predict agronomic performance. In the field, yield depends on stress protection, growth maintenance, reproductive success, recovery capacity, resource-use efficiency, and management context [14,25,26,27,28,29].

The adaptive remodeling framework therefore shifts the translational question. Instead of asking whether a gene is stress inducible, we should ask whether a regulatory module can improve functional performance under realistic stress while minimizing growth cost (Box 1).

Box 1 **Why Single-Gene Stress Tolerance Often Fails in the Field.**

   Many genes that improve stress tolerance under controlled experimental conditions do not consistently enhance field performance. Several factors explain this gap:

1. Controlled stress is not equivalent to field stress. Laboratory treatments are often acute, uniform, and single-factor, whereas field stress is gradual, fluctuating, and combined.
2. Survival is not the same as productivity. A genotype may survive severe stress but show reduced biomass, fertility, quality, or yield.
3. Constitutive stress activation can penalize growth. Continuous activation of ABA signaling, antioxidant defense, osmotic adjustment, or ion transport may consume resources and suppress development.
4. Stress responses are tissue-specific. A gene beneficial in roots may be neutral or harmful in leaves, flowers, fibers, fruits, or seeds.
5. Developmental timing matters. Stress tolerance during seedling growth may not predict reproductive-stage resilience.
6. Genetic background alters network output. The same gene may interact differently with regulatory networks in different cultivars, species, or polyploid genomes.
7. Combined stresses are non-additive. Drought plus heat, salt plus alkalinity, flooding plus reoxygenation, or heavy metals plus microplastics may produce network states that cannot be predicted from single-stress experiments.

   These limitations argue for a shift from single-gene tolerance models toward interaction-centered network tuning and field-relevant phenotyping.

### 6.2. Natural Variation and Multi-Omics Reveal Tunable Modules

Natural variation provides a powerful route for crop improvement because natural alleles have been filtered through evolutionary, ecological, and breeding histories. They often represent balanced modifications of regulatory modules rather than extreme activation of stress pathways [21,23,75,76,78,94].

Stress-related variation may occur in coding regions, promoters, untranslated regions, structural variants, copy-number variants, epigenetic states, or protein-interaction interfaces. Such variation can influence receptor sensitivity, transporter activity, transcription-factor binding, root architecture, stomatal traits, flowering time, or metabolic capacity.

Multi-omics can connect these variants to regulatory architecture. Transcriptomics, proteomics, phosphoproteomics, metabolomics, ionomics, epigenomics, and phenomics reveal different layers of adaptive remodeling [10,30,93,97]. The key challenge is to move from candidate lists to causal modules through time-series data, tissue-specific sampling, perturbation experiments, and field validation. Cotton stress regulators such as GhWRKY46 and GhWRKY207 further illustrate how crop-specific transcriptional modules can contribute to drought, salt, and redox-related stress adaptation [58,98].

### 6.3. Genome Editing and Regulatory Design

Genome editing provides new opportunities to engineer stress resilience, but target choice is critical. Editing terminal stress-responsive genes may improve specific traits but can also produce pleiotropy or growth penalties. A more promising strategy is to tune regulatory nodes that control adaptive remodeling [99,100,101,102,103].

Concrete evidence is currently strongest for promoter, cis-regulatory, and allele-based editing strategies that adjust expression level, tissue specificity, developmental timing, or stress inducibility [101,102,103]. For example, promoter editing has been used to engineer quantitative trait variation in tomato domestication-related traits [101], and CRISPR-Cas9-generated ARGOS8 variants improved maize grain yield under field drought-stress conditions [104]. These examples support regulatory tuning as a practical route for improving stress-related performance while limiting constitutive growth penalties.

By contrast, direct editing of protein-interaction interfaces, phosphorylation sites, degrons, SUMOylation motifs, redox-sensitive residues, or transporter regulatory domains remains an emerging strategy. These targets are conceptually attractive because they may alter stress responsiveness without abolishing normal protein function, but field-validated examples are still limited. Therefore, such edits should be presented as testable precision-tuning strategies rather than as already established crop-resilience solutions [10,44,45,102,103].

Thus, genome editing should be viewed as a tool for network tuning, not simply gene activation or knockout. Edited lines must be evaluated across developmental stages, stress intensities, stress combinations, genetic backgrounds, and field environments.

### 6.4. Dynamic Phenotyping and Field Validation

The success of stress-resilience breeding depends on phenotyping. Traditional assays often measure final injury or survival, whereas resilience is dynamic and includes response, maintenance, recovery, reproductive performance, and yield formation [29,30,31,32,33].

High-throughput phenotyping can measure canopy temperature, stomatal kinetics, root architecture, chlorophyll fluorescence, photosynthetic stability, ion partitioning, reproductive traits, and recovery after stress release [29,30,31,32,33,74]. Machine-learning-based image analysis is also emerging as a useful approach for early stress-tolerance screening, particularly when combined with chlorophyll fluorescence or seedling-growth phenotypes [105]. However, more data do not automatically improve selection. The most valuable phenotypes are those that reflect functional modules and predict field performance.

Field validation should be integrated into stress biology from the beginning rather than treated as a final step. Controlled experiments establish causality, but field trials reveal whether a module improves performance under fluctuating light, temperature, soil moisture, nutrient availability, microbial interactions, and combined stresses [14,25,26,27,28,29,31,32,33].

### 6.5. Designing Crop-Ready Stress-Resilience Modules

A crop-ready stress-resilience module should improve performance under realistic stress without large penalties under favorable conditions. It should act in the appropriate tissue, developmental window, and environmental context, and it should affect agronomically meaningful traits such as yield stability, reproductive success, water-use efficiency, ion homeostasis, recovery, or quality (Box 2).

Promising modules include stomatal regulatory modules, root architecture modules, ion and pH-homeostasis modules, redox and metabolic buffering modules, reproductive protection modules, stress-memory and recovery modules, and stress-inducible promoter modules [12,17,21,23,55,57,78,82,83,99].

These modules should not be optimized independently. Enhancing stomatal closure without maintaining photosynthetic capacity may reduce yield. Increasing root growth without sufficient carbon supply may be costly. Improving ion sequestration without energy balance may suppress growth. Strengthening antioxidant capacity without preserving ROS signaling may impair adaptation. Therefore, the key principle is coordination.

Box 2 **Criteria for a Crop-Ready Stress-Resilience Module.**

    A stress-resilience module is more likely to be useful in breeding or biotechnology if it meets the following criteria:

1. Field relevance: It improves performance under a realistic stress scenario.
2. Conditional activation: It is activated under stress but minimally affects growth under favorable conditions.
3. Tissue specificity: It acts in the organ or cell type where protection is needed.
4. Low growth penalty: It enhances resilience without excessive reduction in biomass, fertility, quality, or yield.
5. Recovery benefit: It improves post-stress recovery, not only survival during stress.
6. Reproductive protection: It maintains flowering, pollination, seed set, fruit set, fiber quality, or grain filling.
7. Compatibility with breeding: It can be selected, edited, introgressed, or phenotyped reliably.
8. Stability across environments: It remains beneficial across stress intensities, seasons, locations, or management systems.

## 7. Conceptual Framework: Intermolecular-Interaction-Driven Adaptive Remodeling

Plant abiotic stress tolerance has often been described in terms of individual genes, signaling pathways, or physiological traits. This approach has been highly productive, leading to the identification of ABA receptors, SnRK2 kinases, PP2C phosphatases, ROS-producing and ROS-scavenging enzymes, Ca^2+^ sensors, transcription factors, ion transporters, heat-shock proteins, osmoprotectant pathways, chromatin regulators, and stress-associated metabolites [1,2,3,4,5,7,8,9,12]. However, component-centered models alone cannot fully explain how plants generate coordinated adaptive responses across tissues, developmental stages, stress combinations, and field environments.

We define intermolecular-interaction-driven adaptive remodeling as the process by which stress-induced changes in molecular interactions reorganize regulatory networks and generate adaptive physiological states, as summarized in Figure 1. These interactions include hormone–receptor binding, peptide–receptor recognition, protein–protein association, Ca^2+^–sensor binding, redox-sensitive protein modification, phosphorylation, phosphatase-mediated attenuation, transcription factor–DNA binding, chromatin-associated regulation, transporter–ion interaction, and metabolite-mediated feedback [3,5,8,10,11,18,36,44,45,61,62,63,64].

This framework emphasizes that stress tolerance is not a static property of a genotype or the direct consequence of a single tolerance gene. Rather, it is an emergent property of network behavior. A regulatory component may enhance tolerance in one context but reduce fitness in another because its output depends on interacting partners, expression domain, developmental stage, stress intensity, metabolic status, and environmental combination [2,13,14,25,26,27,28]. Thus, the adaptive value of a component depends on how it is embedded within a larger interaction network.

A central feature of adaptive remodeling is functional selection under resource limitation. Plants rarely optimize all functions simultaneously during stress. Water conservation, carbon gain, ion homeostasis, redox control, protein protection, reproductive development, and recovery may compete for limited energy and resources. These trade-offs are not failures of stress response; they are adaptive choices shaped by network state, environmental severity, developmental priority, and resource availability [17,21,71,86,87,88,89,90,91].

The framework also explains stress specificity. Many stresses activate overlapping hubs such as ABA, ROS, Ca^2+^, kinases, transcription factors, transporters, and metabolites, but outputs differ because initial disturbances, signal dynamics, tissue context, and interaction partners differ [1,2,5,8,12]. Therefore, stress specificity should be understood as a property of network configuration rather than as the action of stress-exclusive components.

This perspective has practical implications. Traditional strategies often focus on identifying and manipulating stress-responsive genes, but field performance is frequently limited by pleiotropy, growth penalties, tissue nonspecificity, developmental mismatch, and environmental dependence [29,30,31,32,33]. A more effective strategy is to tune regulatory modules. Promoter editing, allele replacement, phosphorylation-site editing, protein-interaction interface modification, transporter-domain tuning, and tissue-specific expression design may adjust network behavior without abolishing normal function [10,21,78,100,101,102,103,105].

The adaptive remodeling model generates testable predictions (Box 3). Stress-resilient genotypes should show greater network flexibility and recovery capacity than sensitive genotypes. Editing regulatory interfaces or promoter responsiveness may produce more balanced phenotypes than constitutive overexpression. Cell-type-specific network rewiring should explain why the same component produces different outcomes in roots, guard cells, vascular tissues, meristems, and reproductive organs [52,53]. Combined stresses should generate emergent network states that cannot be predicted from single-stress responses alone [14,25,26,27,28]. Stress memory may involve partial stabilization of interaction networks through chromatin, metabolic, or protein-level feedback [11,60,61,62,63,64].

Therefore, intermolecular-interaction-driven adaptive remodeling is not merely a descriptive concept. It provides a framework for designing experiments, interpreting multi-omics data, identifying crop-ready regulatory modules, and linking molecular mechanisms to field resilience. Figure 1 integrates this logic by linking abiotic stress inputs, interaction switching, signaling hubs, functional outputs, and crop-level resilience.

Abiotic stresses, including drought, salinity, alkalinity, heat, cold, flooding, heavy metals, nutrient imbalance, and emerging pollutants, disturb plant homeostasis through osmotic, ionic, redox, metabolic, structural, and developmental constraints. These disturbances induce rapid molecular interaction switching, including receptor–ligand recognition, protein–protein interaction, Ca^2+^-sensor binding, redox-sensitive modification, phosphorylation and dephosphorylation, protein–DNA binding, chromatin-associated regulation, transporter–ion interaction, and protein–metabolite feedback. These interaction changes converge on core signaling hubs such as ABA, ROS, Ca^2+^, kinase/phosphatase networks, transcription factors, transporters, metabolic regulators, and chromatin-associated factors. In this model, ROS are not treated solely as damage-associated molecules, but as redox switches that integrate stress perception, defense activation, senescence-related transitions, systemic signaling, and recovery. Chromatin-associated mechanisms may further stabilize or reset stress-induced network states during priming and stress memory. Through network remodeling, plants generate adaptive outputs including stomatal adjustment, root architecture remodeling, ion and pH homeostasis, antioxidant defense, metabolic reprogramming, structural protection, stress memory, reproductive resilience, and post-stress recovery. The model emphasizes that plant stress tolerance is not the direct consequence of single stress-responsive genes, but an emergent property of dynamic interaction networks that rebalance growth, defense, reproduction, and resource allocation under fluctuating environments.

Box 3 **Testable Predictions of the Adaptive Remodeling Model.**

   The intermolecular-interaction-driven adaptive remodeling framework generates several experimentally testable predictions:

1. Stress resilience should correlate more strongly with network flexibility and recovery capacity than with maximal expression of individual stress-responsive genes.
2. Editing promoter responsiveness, transporter regulatory domains, degrons, phosphorylation sites, or protein-interaction interfaces represents a testable strategy for tuning network behavior; however, protein-level precision editing requires rigorous validation under field-relevant stress conditions.
3. Cell-type-specific network rewiring should explain why the same signaling component has different effects in roots, guard cells, vascular tissues, meristems, and reproductive organs.
4. Combined stresses should produce emergent interaction states that cannot be predicted by simply adding single-stress responses.
5. Stress memory should involve partial stabilization of interaction networks through chromatin remodeling, metabolite feedback, altered signaling thresholds, or protein-level regulation.
6. Crop resilience should depend on maintenance and recovery of functional modules, including stomatal responsiveness, root acquisition capacity, ion partitioning, reproductive stability, and yield formation, rather than survival alone.

   These predictions provide a practical route for converting the adaptive remodeling framework into experimentally testable research programs.

## 8. Open Questions and Future Directions

The adaptive remodeling framework highlights several unresolved questions.

First, how are abiotic stresses initially perceived at the cell surface and cell wall–plasma membrane interface? Downstream pathways involving ABA, ROS, Ca^2+^, kinases, and transcription factors have been extensively studied, but the earliest steps of stress perception remain incompletely understood for many stresses [1,2,37].

Second, how do plants generate specificity from shared signaling hubs? ABA, ROS, Ca^2+^, MAPKs, CDPKs, CBL–CIPK modules, and transcription factors participate in multiple stress responses, yet drought, salt, heat, cold, flooding, and heavy metals produce distinct physiological outputs [2,5,8,9,12].

Third, how does stress signaling differ among cell types? Bulk transcriptomic or proteomic analyses can obscure distinct responses in root epidermis, cortex, endodermis, vascular tissues, guard cells, meristems, and reproductive organs. Single-cell and spatial approaches are beginning to reveal how cell identity shapes stress responses [52,53].

Fourth, how do plants balance stress protection with growth and reproduction? Many protective mechanisms require energy, carbon skeletons, reducing power, or nutrient redistribution. Excessive activation of defense pathways may reduce photosynthesis, biomass accumulation, flowering, seed set, or grain filling [13,17,86,87,88,89,90,91].

Fifth, how stable and useful is stress memory? Stress priming and memory have been observed at physiological, transcriptional, epigenetic, chromatin-associated, and metabolic levels, but it remains unclear which memory states are transient, stable, reversible, heritable, or associated with yield stability under field conditions. Future work should distinguish short-term priming from durable memory and should determine whether specific epigenome or chromatin modifications improve recovery and reproduction without excessive growth cost [60,61,62,63,64].

Sixth, how can mechanistic discoveries be validated under realistic field-like stress combinations? Most mechanistic studies examine single stresses under controlled conditions, whereas crops experience fluctuating combinations of water deficit, heat, salinity, alkalinity, nutrient imbalance, pathogen pressure, and pollutant exposure [14,25,26,27,28]. Future research must integrate controlled mechanistic experiments with field phenotyping, recovery assays, reproductive-stage exposure, and predictive modeling [29,30,31,32,33].

Addressing these questions will require plant stress biology to move from component discovery toward interaction-centered, cell-type-resolved, time-resolved, and field-relevant analysis.

## 9. Conclusions

Plant abiotic stress responses are governed by dynamic interaction networks rather than isolated linear pathways. As summarized in Figure 1, ABA, ROS, Ca^2+^, kinase/phosphatase systems, transcription factors, transporters, metabolites, chromatin regulators, membranes, organelles, and structural components form interconnected modules that perceive stress, decode environmental information, and reorganize plant function [1,2,3,5,8,9,13]. These modules are reused across different stresses, but their outputs depend on interaction context, tissue specificity, developmental stage, signal dynamics, and environmental combination.

The concept of intermolecular-interaction-driven adaptive remodeling provides a unifying framework for understanding this complexity. It emphasizes that stress adaptation emerges from changes in molecular interactions and regulatory network topology. Through adaptive remodeling, plants select functional states that balance water conservation, ion homeostasis, redox control, metabolic stability, structural protection, growth, reproduction, and recovery. This perspective helps explain why the same signaling components can generate different outcomes under different stresses, why tolerance mechanisms often involve growth costs, and why single-gene strategies frequently fail to deliver stable field performance [14,25,26,27,28,29].

The future of plant stress biology will depend on moving from component discovery to interaction-centered models that explain how plants select adaptive states under fluctuating environments. By focusing on molecular interactions, regulatory networks, physiological modules, and crop-level traits, researchers can better connect mechanistic discoveries with field resilience. This perspective provides both a conceptual advance for stress biology and a practical foundation for designing crops that maintain productivity, quality, and recovery capacity under increasingly variable environments.

## Data Availability

No new experimental data were generated in this review. The literature analyzed in this review is available from the cited publications.
